# Diagnosis and Treatment of Leprosy in Taiwan during the COVID-19 Pandemic: A Retrospective Study in a Tertiaty Center

**DOI:** 10.3390/diagnostics13243655

**Published:** 2023-12-13

**Authors:** Chin-Ling Hsieh, Pa-Fan Hsiao

**Affiliations:** 1 Department of Dermatology, MacKay Memorial Hospital, Taipei 10449, Taiwan; jeff415623@gmail.com; 2 Department of Medicine, MacKay Medical College, New Taipei City 25245, Taiwan; 3 Department of Cosmetic Applications and Management, MacKay Medicine, Nursing and Management College, Taipei 11260, Taiwan

**Keywords:** leprosy, Taiwan, COVID-19

## Abstract

Currently, over 200,000 new cases of leprosy are reported annually worldwide. Although leprosy was thought to have been eradicated in Taiwan, a few new cases still occur annually. Protean clinical manifestations of leprosy and immunological reactions result in delayed diagnoses. In addition, drug-resistant leprosy is emerging and poses treatment challenges. In this retrospective study, we collected and analyzed the clinicopathological features, leprosy type, treatment response, and relapse rate of patients with leprosy in our hospital between January 2009 and November 2022. We found that 54% of patients were Indonesian, and borderline lepromatous leprosy was predominant (39%); moreover, histoid leprosy and the Lucio phenomenon were also reported. Polymerase chain reaction analysis identified four positive cases, including a dapsone-resistant (4%) case. Our findings indicated good control of leprosy and a lower rate of dapsone resistance than that reported by the World Health Organization (4% vs. 13%) from 2009 to 2015. We found that the patient profile in terms of the treatment duration, recurrence rate, systemic symptoms, and neurological symptoms did not differ between before and during the pandemic. We report the recent advances in leprosy diagnosis, drug-resistant gene mutations, post-exposure prophylaxis, vaccination, and the effect of coronavirus disease 2019 on leprosy to facilitate updated leprosy diagnosis and management.

## 1. Introduction

Leprosy, or Hansen’s disease, results from *Mycobacterium leprae* (*M. leprae*) infection and affects the skin, peripheral nerves, eyes, and mucosa, leading to permanent deformities if untreated. According to the World Health Organization (WHO) weekly report, 140,594 new cases were reported globally in 2021, with more than half in the South East Asia region (66%) [1]. Despite meeting the eradication criteria set by the WHO (prevalence rate < 1 patient per 10,000), Taiwan records approximately 10 new cases annually [2]. Based on the Ridley and Jopling classification spectrum [3], leprosy is classified into the tuberculoid type (TT) or the lepromatous type (LL). Leprosy can also be classified into paucibacillary (PB) and multibacillary (MB) types. Multi-drug therapy (MDT), consisting of rifampicin, dapsone, and clofazimine, was introduced by the WHO in 1982 to control leprosy. Patients with MB and PB can be treated with MDT for 12 and 6 months, respectively. Drug resistance has been reported since 1964 and has gradually increased despite MDT [4]. Drug resistance and relapse remain major public health issues in the control of leprosy. The following tools have been developed for drug resistance surveillance: in vivo methods using Shepard’s mouse footpad model, molecular testing including polymerase chain reaction (PCR) sequencing, and whole-genome sequencing (WGS). New molecular techniques are promising alternatives for the detection of antimicrobial resistance in leprosy, although improvements are required in the methodological quality of these techniques [5].

The coronavirus disease 2019 (COVID-19) outbreak raises further uncertainty regarding the diagnosis of leprosy and its treatment. Leprosy reactions can be triggered by COVID-19 vaccination [6]; however, the overall effect of COVID-19 on the clinical course and treatment of leprosy remains unclear. Studies on leprosy, particularly considering the COVID-19 pandemic, have not been conducted recently in Taiwan. Therefore, this study aimed to collect and analyze the clinicopathological features, leprosy type, treatment response, and relapse rate of patients with leprosy in our hospital.

## 2. Materials and Methods

This single-center retrospective study included 28 patients diagnosed with suspected leprosy between January 2009 and November 2022. Four hospitals in Taiwan specialize in diagnosing and referring patients with leprosy: two in northern Taiwan, one in central Taiwan, and one in southern Taiwan. Our hospital is situated in northern Taiwan. Clinical and histopathological data were obtained from medical records in the dermatopathology database of the hospital. Leprosy diagnosis was based on clinical manifestations, physical examination, slit-skin smears, and skin lesion biopsies. Two reviewers (P.-F.H and C.-L.H) obtained pathologic slides from the Dermatopathology Department and reviewed the pathologic findings of the slides. The reviewers’ individually recorded decisions were compared, and disagreements were resolved. PCR was performed, and the DNA samples extracted from the paraffin blocks were subjected to PCR amplification using primers for *M. leprae*. The forward primer was 5′-CTCGACGATCAAGCTGAGAC-3′, and the reverse primer was 5′-CACCTAGCACGTCCTCCAAT-3′ [7]. As for drug resistance, *M. leprae*-specific repetitive sequences (RLEP) were used in the PCR to detect *M*. *leprae* DNA for facilitating the diagnosis of PB leprosy and surveying drug-resistant target genes, such as *folp1*, *rpoB*, and *gyrA*, which are related to dapsone, rifampin, and ofloxacin resistance, respectively. The clinical characteristics included age at diagnosis, sex, Ridley–Jopling classification [8], leprosy reaction, bacterial index of slit-skin smear, skin lesions, peripheral nerve damage, drug resistance with associated genes, and treatment. The pathological data included granuloma formation, neuritis, vasculitis, acid-fast staining (AFS), and PCR analysis for *M*. *leprae*.

Descriptive statistics using means, percentages, standard deviations, and frequencies were performed. Data were analyzed using SPSS version 19.0 (IBM SPSS Statistics for Windows, Version 19.0. Armonk, NY, USA: IBM Corp.). The precision of the effect size was reported as a 95% confidence interval (CI). Statistical analyses were performed on data from patients who completed therapy. A chi-square test was used to determine differences in the recurrence rates, systemic symptoms, and neurological symptoms between before and after the pandemic started in Taiwan (i.e., in 2020). An independent *t*-test was used to evaluate the impact of COVID-19 on the treatment duration. Statistical significance was set at *p* < 0.05 for both the chi-square and independent *t*-tests.

This study was approved by the Institutional Review Board of Mackay Memorial Hospital (23MMHISO66e). Owing to the de-identification of patient information and the retrospective nature of the study, written informed consent was not obtained.

Relevant studies published between January 2020 and May 2023 were identified using PubMed. The Medical Subject Headings term “leprosy” was used. Articles related to drug resistance, biomarkers, molecular diagnosis, the impact of COVID-19 on leprosy, vaccines, and post-exposure analysis were selected from the search results. The search was expanded using the “related articles” option in PubMed, and all abstracts, studies, and citations retrieved were reviewed. In addition, other studies were identified using the reference sections of the relevant papers and corresponding subject experts. Language restrictions were not imposed. A literature review was conducted to update our knowledge of drug resistance, molecular diagnosis, and the association between COVID-19 and leprosy.

## 3. Results

Our hospital reported 28 cases of leprosy between 2009 and 2022. Patients had an average age of 38 years, and 65% were female. Five (18%) Taiwanese cases were noted in our study, and the majority were from Indonesia (54%). Most patients (86%) had multiple cutaneous lesions. Clinical classification included LL (18%), borderline LL (39%), borderline borderline (11%), borderline TT (18%), and TT (4%). Among patients from foreign countries, borderline LL was the most common clinical classification, whereas patients in Taiwan were primarily indeterminate. We observed two indeterminate and one histoid case. 

**Figure 1:**
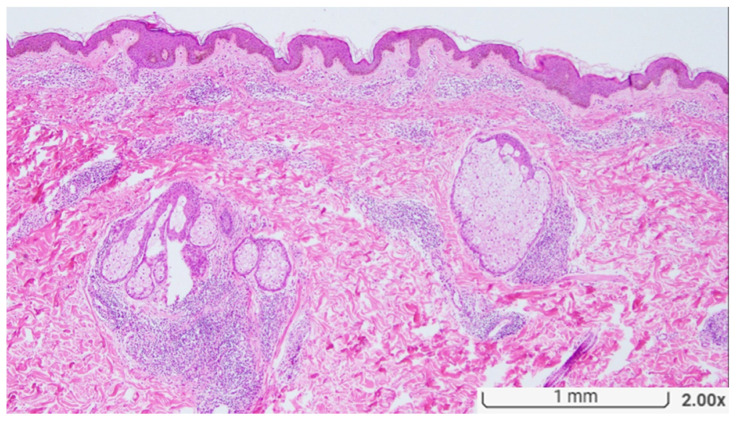
Histopathologically, there is a dense perivascular infiltration with scattered granulomas in both the upper and deep dermis. The granulomas are edematous and loosely formed. Small nerves surrounded by the granulomatous inflammation are focally seen. (Original magnification, H&E ×20).

Nine patients presented with active leprosy reactions. Approximately 18% (*n* = 5) of our patients had type I reactions (Figure 1), three had type II, and one had type III. Of these, one 32-year-old woman from Indonesia experienced a type III reaction that presented with erythematous swelling patches and hemorrhagic bullae on the bilateral lower legs, feet, and toes, a condition known as the Lucio phenomenon [9]. Intermittent fever was noted during the treatment course. This patient was treated with MDT and systemic prednisolone, and symptoms improved.

Among the 27 patients who underwent slit-skin smear examinations, 15 (54%) had positive bacterial indices. Thirty-two percent (*n* = 9) of our patients had peripheral nerve involvement with neurological symptoms such as numbness, loss of sensation, muscle weakness, and palpable nerves. Two patients experienced joint pain and muscle atrophy. No symptoms of eye involvement were observed; however, one patient had bony destruction with a Charcot foot. Table 1 summarizes the clinical characteristics of patients (details in Table S1).

**Figure 2:**
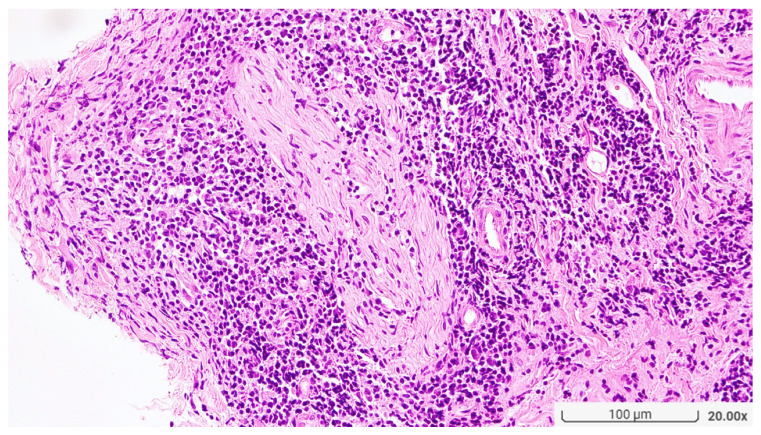
Small nerve bundles are surrounded by granulomatous infiltration. (Original magnification, H&E ×200).

Among the 34 specimens that were subjected to pathological examinations, 76% (*n* = 26) had granuloma, 65% (*n* = 26) had neuritis (Figure 2), and 9% (*n* = 3) had vasculitis. These specimens displayed inflammatory cell infiltration, including lymphocytes, foamy histiocytes, and plasma cells.

One case of histoid leprosy showed diffuse histiocytic infiltration throughout the reticular dermis. The cells tested negative for S-100. Numerous plasma cells and globi were observed. AFS showed numerous bacilli within the specimens [10]. In a type III reaction, necrotic vessel walls with perivascular infiltration and nuclear dust were observed, along with lobular panniculitis with globi [9]. Numerous acid-fast bacilli were found in endothelial cells and associated with vasculitis. 

Regarding pathological diagnostic markers, positive AFS results were reported in 25 specimens (74%). PCR analysis for *M. leprae* was performed on nine samples, of which four showed positive results. Drug-resistant gene detection was performed on four specimens via PCR analysis, and a mutation in *folp1* was reported. In contrast, no mutations were detected in *rpoB* or *gyrA*. Pathological features of the specimens are summarized in Table 2 (details in Table S2).

Seven patients were diagnosed with leprosy during the COVID-19 pandemic (Table S3). Fourteen patients completed the treatment; the average duration of treatment was 17.8 months. Three cases of recurrences were noted. However, no significantly longer treatment durations (*p* = 0.318) and increased rates of recurrence (*p* = 0.923), systemic symptoms (*p* = 0.425), and neurological symptoms (*p* = 0.347) were observed in the patients during the pandemic.

## 4. Discussion

The patient profile in our hospital reflects the present status of patients with leprosy in Taiwan, which comprises primarily foreigners or immigrants and a limited number of local residents. The clinical course and treatment were not affected by COVID-19 outbreaks. Borderline LL was diagnosed in 39% of the patients, and histoid leprosy was also observed. Neurological symptoms were recorded in 32% of patients; the predominant pathological findings were granuloma and neuritis in 76% and 65% of cases, respectively. PCR analysis revealed four positive cases of *M*. *leprae*, and drug-resistant genes were detected. During the COVID-19 pandemic, seven patients were diagnosed with leprosy at our hospital, with no patients contracting COVID-19 during the study period. Leprosy reactions have been reported to develop 1–2 weeks after COVID-19 vaccination [11,12]. Four patients with type I reactions and one patient with type II reactions were identified in the present study; however, these patients denied the occurrence of skin lesions 1–2 weeks after receiving the COVID-19 vaccine. Thus, our study demonstrated no association between COVID-19 vaccination and the incidence of leprosy. Because there were no patients with COVID-19, our data were insufficient to investigate the impact of leprosy treatment on the severity of COVID-19.

### 4.1. Molecular and Serological Diagnoses

Early detection of *M*. *leprae* is crucial to prevent transmission and reduce permanent nerve damage and physical disabilities. Current diagnosis is based on clinical examinations, with slit-skin smears widely used by clinicians. Preferred sampling sites include earlobes, contralateral eyebrows, mucosa, forehead, chin, elbows, knees, and dorsal surfaces of fingers, with a high probability of detecting positive bacilli [13]. The specificity of slit-skin smears can reach 100%; however, a 5-year sensitivity of 34% has been reported [14]. Skin biopsies aid in confirming leprosy and classifying its type based on the Ridley and Jopling classification. Histopathological examination demonstrates approximately 70% specificity and sensitivity. In PB and early-stage leprosy, the history and clinical findings may be inconclusive and histopathological findings are non-specific. Owing to low sensitivity, slit-skin smear requires a minimum of $10^{4}$ bacilli per gram for detection under microscopy. These limitations pose diagnostic challenges. However, molecular and serological diagnostic tools have been developed with improved sensitivity and specificity. 

PCR analysis of slit-skin smears and skin biopsy specimens has been used since 1990 to detect *M*. *leprae* [15]. The following sequences, including gene regions encoding *M*. *leprae* 18-, 36-, and 65-kDa antigens, 16S rRNA subunit, and *M*. *leprae*-specific repetitive sequences (RLEP), are used for PCR [16]. RLEP is the preferred target because of its high copy number; however, 16S rRNA shows higher specificity [17]. Specimens fixed in 10% neutral buffered formalin or 50% or 70% ethanol are commonly used for PCR detection of *M*. *leprae* DNA. However, the fixation method of the specimen affects DNA amplification. Ethanol fixation is presumed superior to formalin fixation [18]. Real-time PCR (RT-PCR) enhances speed, cost, and sensitivity compared to conventional PCR, requiring approximately 2.5 h to detect DNA. Nested PCR was developed for whole blood detection of *M*. *leprae* DNA, with no significant differences between quantitative PCR (qPCR) and nested PCR in patients diagnosed with PB [19]. For patients with MB, sensitivity and specificity of 87.00% and 93.30%, respectively, were reported for qPCR. A meta-analysis in 2021 demonstrated similar specificity (93.60%) but lower sensitivity (47.50%) [20]. 

Recently, a loop-mediated isothermal amplification assay (LAMP) was developed as a simpler alternative to PCR, especially for field use. Furthermore, the LAMP offers straightforward DNA extraction and visible amplicon detection [21]. Although LAMP has lower sensitivity than qPCR for skin biopsy specimen analysis, it has similar sensitivity (84%) and specificity (100%) to qPCR for the detection of *M*. *leprae* in slit-skin smears, reducing invasive tissue sampling and providing a rapid, field-friendly, and cost-effective examination for clinicians [22]. 

Phenolic glycolipid 1 (PGL-I), an *M*. *leprae*-specific antigen, was first discovered in 1981. Serological tests for detecting IgM anti-PGL-I antibodies have been used for the diagnosis of leprosy and monitoring treatment effects. An enzyme-linked immunosorbent assay is the most commonly used technique for detecting anti-PGL-I antibodies, despite other methods, such as dipstick and lateral flow immunochromatographic assays, being simpler for anti-PGL-I antibody detection [16]. Serological testing is based on the antibody response, which is less effective in detecting patients with PB. A sensitivity of 63.8% and specificity of 91% have been reported for anti-PGL-I serological detection [23]. 

For diagnosis, we performed PCR analysis in suspected patients without clear pathological results, negative AFS, or low or negative bacterial indices. Negative PCR results in patients diagnosed with leprosy may be due to the presence or absence of bacteria in specimens. All specimens in our study were fixed in formalin, embedded in paraffin, and subjected to histopathological and PCR analyses. Although *M*. *leprae* DNA is detectable in formalin-fixed tissues, formalin sometimes compromises DNA amplification. Therefore, the choice of fixative may affect PCR analysis results, and future studies should consider splitting specimens. To acquire better quality DNA amplification, half of the specimen should be fixed in formalin and sectioned for histopathology, and the other half fixed in 50% or 70% ethanol for PCR analysis [18].

### 4.2. Drug Resistance

Dapsone, used to treat leprosy since 1940, competes with p-aminobenzoic acid and targets dihydropteroate synthase, which is encoded by *folp1*, thus interfering with folic acid biosynthesis. However, a dapsone-resistant strain of *M*. *leprae* was first detected in 1964; consequently, the WHO introduced MDT, which comprises dapsone, rifampicin, and clofazimine, as a first-line medication in 1982 [24]. Minocycline, fluoroquinolones, and clarithromycin are second-line medications that are not included in the WHO recommendations. Rifampicin is the primary bactericidal component of MDT. Rifampicin binds to the β subunit of RNA polymerase, which is encoded by *rpoB*, consequently disrupting messenger RNA (mRNA) transcription [25]. Clofazimine is also a bactericidal component of MDT; however, its antimicrobial mechanism has not been fully investigated. A possible mechanism against leprosy is disruption of the membrane structure and function of *M*. *leprae* or increasing phospholipase A2 activity and production of an enzymatic hydrolysis product. Fluoroquinolones such as pefloxacin and ofloxacin have been used as alternatives or additional agents for MDT. Ofloxacin (4-fluoroquinolone), an effective alternative regimen, targets the gyrA subunit of DNA gyrase A, which is encoded by *gyrA*, thus interfering with DNA replication; it achieved a 99.9% bacterial clearance after four weeks of administration in a phase III trial [26]. Minocycline, the sole tetracycline agent, has demonstrated bactericidal activity against *M*. *leprae* [27]. Minocycline binds to the 30S subunit of the bacterial ribosome and interrupts the binding of transfer RNA to mRNA, thereby blocking the translation of bacterial proteins. Minocycline showed faster bacterial clearance than fluoroquinolones. Minocycline and ofloxacin had a similar efficacy to 2 years of dapsone and clofazimine and showed significant efficacy against rifampicin-resistant strains, with fewer side effects and improved patient compliance [28]. However, relapse rates associated with these agents require further investigation. Clarithromycin is a macrolide antibiotic that binds to the 50S subunit of the bacterial ribosome and interferes with the translation of bacterial proteins. Clarithromycin showed similar efficacy against *M*. *leprae* as minocycline [25]. Clarithromycin (2000 mg) is as effective as three months of rifampicin [28]. In our study, all patients completed MDT according to the WHO guidelines for leprosy treatment [29]. Rifampicin, ofloxacin, and multi-drug resistance were not observed in our study. Drug resistance to dapsone was 4%, notably lower than the 13% reported by the WHO surveillance network from 2009 to 2015. Similarly, the rate of drug resistance reported in our study was lower than that (11.43%) demonstrated in a systematic review and meta-analysis in South East Asia in 2022 and that in Taiwan (11.1%, *n* = 3) between 2013 and 2016 [2]. 

Drug resistance is typically associated with poor compliance, treatment irregularities, inappropriate medication, and long-term treatment. Mutations in codons 53 (Thr) and 55 (Pro) of *folp1* induce drug resistance to dapsone [30]. Mutation analysis revealed that Pro55 was most frequently replaced with Leu, followed by Arg. Mutations in codons 438, 440, 441, 456, 458, and 460 of *rpoB* confer resistance to rifampicin. The highest frequency of mutations was observed for the substitution of Ser450 with Leu. Mutations in codons 89 and 91 of *gyrA* are associated with ofloxacin resistance. The replacement of Ala with Val was the foremost mutation pattern. The development of drug resistance against clofazimines in *M*. *leprae* has rarely been reported. As no direct target gene has been identified, it is difficult to identify a clofazimine-resistant strain. Furthermore, minocycline resistance has not yet been reported.

*M*. *leprae* cannot be cultured in vivo. In 1964, a mouse footpad assay was developed to examine drug susceptibility. An average of 6–12 months is typical for obtaining results with this method, which requires highly skilled laboratory staff and is not applicable to numerous strains. Therefore, PCR analysis is recommended for the detection of drug resistance, as it is cost-effective and provides results within a day [31]. 

The single case of dapsone resistance in our study occurred in a patient diagnosed with leprosy 50 years ago, whereupon dapsone monotherapy was prescribed (between 1965 and 1967). However, when new lesions developed over three months, the patient had self-medicated and discontinued medication for approximately 6 months. Therefore, drug resistance in this patient was likely related to treatment irregularities and dapsone monotherapy. We performed PCR analysis for drug resistance in patients with suspected therapeutic failure, poor compliance, or relapse. The failure of PCR amplification can be attributed to negative results, which are usually related to a low count or absence of bacteria or PCR inhibitors in the skin [24]. Our study revealed the presence of well-controlled patients at our hospital. Given Taiwan’s leprosy eradication status and the predominantly foreign patient population in our study, a lower drug resistance rate is expected compared to previous studies. As a result, routine examination of drug-resistant genes is not suggested.

### 4.3. Treatment for Leprosy Reactions

Leprosy reactions account for 8–33% of leprosy cases and can be classified into type I and type II reactions. Type I reactions can affect any type of leprosy, whereas type II reactions typically occur in patients with LL. Type I reactions are related to overreactive CD4+ T-cell-mediated cellular responses, whereby macrophages are stimulated by T cells and release cytokines, such as TNF-α, IFN-γ, IL-1β, IL-6, lysozymes, and reactive oxygen species, that induce systemic inflammation. Type II reactions are associated with immune complex deposition, an increased CD4/CD8 ratio, and an elevated neutrophil count; these neutrophils subsequently produce TNF-α, IFN-γ, IL-2, IL-6, and IL-12 [32]. Type I reactions present as erythema of existing skin lesions. Neuritis, fever, skin ulcerations, and arthralgia can occur as severe reactions. Type II reactions manifest as painful erythematous nodules and constitutional symptoms, such as fever, arthritis, neuritis, and proteinuria [33]. Systemic steroids, thalidomide, and immunosuppressants are essential medications for the management of leprosy. Systemic prednisolone (40–60 mg) should be initiated for the management of type I reactions and tapered gradually; methotrexate is recommended as a steroid-sparing agent. Cyclosporine is an effective treatment for severe type I reactions [34] and can serve as an alternative treatment. Systemic steroids, thalidomide, methotrexate, and clofazimine are commonly used to treat type II reactions. Minocycline can be used as a steroid-sparing agent for the management of type II reactions because of its antimicrobial and immunomodulatory effects [35]. Azathioprine is an effective steroid-sparing agent against type I and II reactions [36]. Azathioprine is metabolized into mercaptopurine and 6-thioguanine, which subsequently block purine synthesis, leading to a reduction in peripheral T and B lymphocytes. Long-term steroid usage-associated toxicity, teratogenic effects of thalidomide, skin pigmentation, gastrointestinal side effects of clofazimine, and high risk of myelotoxicity of azathioprine have been reported during treatment. Moreover, precipitation of type II reactions by minocycline has been documented [37]. Thus, owing to the long-term treatment duration and side effects of the current regimen, new agents are required. Apremilast, a selective phosphodiesterase-4 inhibitor, increases intracellular cyclic adenosine monophosphate levels in Th1 and Th17 cells, resulting in a decrease of inflammatory cytokines such as IFN-γ, IL-2, IL-6, and IL-12 and an increase in IL-10 [37]. Administration of apremilast for less than 4 weeks showed significant efficacy against type II reactions in a prospective pilot study [36]. Lower costs and lack of immunosuppression risks make apremilast a promising option for controlling type II reactions [36]. Systemic steroids are still the primary medication used to treat leprosy reactions, and their appropriate use was established in the era of the COVID-19 pandemic. 

### 4.4. Impact of the COVID-19 Pandemic

Leprosy reactions are primarily triggered by MDT initiation. However, pregnancy, infections, stress, and vaccinations can also induce leprosy reactions. For example, influenza, pneumococcal, Bacillus Calmette and Guérin (BCG), and hepatitis B vaccines have all been shown to trigger leprosy. Recently, cases of COVID-19 vaccine-induced type I and type II leprosy reactions have been reported, including the BNT, Moderna mRNA-1273, and ChAdOx1 nCoV-19 vaccines. The average interval between vaccination and leprosy reactions ranged from 5.1 to 11.5 d [38]. Skin biopsy specimens from patients with type II reactions show significant perivascular neutrophilic infiltration throughout the dermis, indicating that neutrophils play a key role in the pathogenesis of type II reactions. Neutrophil infiltration in lung tissue and an elevated neutrophil-to-lymphocyte ratio have been noted during COVID-19 infection [39]. The glycoprotein-S of the COVID-19 vaccine stimulates the release of TNF-α, IL-8, and IFN-γ [40]. Therefore, neutrophil activation and increased TNF-α, IL-8, and IFN-γ are hypothesized to induce type II reactions. 

Leprosy reactions accompanied by acute foot drop have been documented, raising the possibility of neuritis following COVID-19 vaccine injection [38]. While case reports and series have noted leprosy reactions and neurological complications from COVID-19 vaccines, it is important to emphasize that patients should still receive the COVID-19 vaccine for prevention. Nonetheless, patients should be informed of the side effects and possible complications associated with leprosy reactions and neuritis. Prednisolone causes immunosuppression in a dosage >10 mg/d or a total cumulative dosage >700 mg [41]. The use of systemic steroids places patients at higher risk of severe COVID-19 infection and complications. However, an increased risk of COVID-19 in patients taking systemic steroids has not been demonstrated. The development of severe COVID-19 complications was not observed in patients taking systemic steroids for the management of leprosy [42]. The use of systemic steroids, thalidomide, and dapsone does not affect the occurrence or severity of COVID-19 [43]. Therefore, systemic steroids should be used for the recommended indications. During the pandemic, MDT and an optimal dose of systemic steroids were preserved, and prednisolone < 20 mg/d was preferred. Our treatment strategy also followed these suggestions. Immunosuppressive agents, such as methotrexate, azathioprine, and cyclosporine, should be avoided or prescribed under close monitoring [44].

### 4.5. Leprosy Prevention

Vaccines have been developed for leprosy prevention. The BCG vaccine, initially used for the prevention of *M*. *tuberculosis*, has been implemented against leprosy, with protective effects in the range of 26–41% [28]. However, the efficacy declined over time and showed better results in women [45]. Antigens from the Indian Cancer Research Center (ICRC) bacillus revealed cross-reactivity with *M*. *leprae* and can be administered as a single dose at a sustainable antibody level [46]. The combination of the ICRC bacillus and BCG vaccines showed superior efficacy compared with BCG alone. The *M*. *indicus pranii* vaccine demonstrated beneficial effects by extending recovery time and increasing antimicrobial effects [30]. LepVax is a recombinant protein vaccine consisting of *M*. *leprae* antigens ML2531, ML2380, ML2055, and ML2028 (LEP-F1) and synthetic Toll-like Receptor 4. LepVax delayed neuropathy and onset of nerve conduction deficits in animal studies [47,48]. Moreover, the safety and immunogenicity of LepVax have been demonstrated in a phase 1a trial [47].

To prevent leprosy, post-exposure prophylaxis has been studied for high-risk individuals, such as household contacts. Initially, dapsone and acedapsone showed an overall protective effect of 60% in a meta-analysis comprising 14 trials conducted in 2000 [49]. Owing to long-term administration, associated drug resistance, and poor compliance, newer agents were considered. The effects of rifampin were investigated in a randomized controlled trial. A single dose of rifampin or placebo was prescribed to close contacts in this study, showing an overall protective effect of 57% compared with the placebo. However, no significant difference between rifampin and placebo was observed two years after administration [50]. In 2023, another trial compared rifapentine, rifampin, and no intervention for leprosy prevention in close contacts. After 4 years, the rifapentine group had an 84% lower leprosy incidence than the control group, whereas no significant difference was found between rifampin and the control [51]. This study showed that the protective effects of rifapentine were greater than those of rifampin. Nonetheless, both drugs hold the promise for post-exposure prophylaxis. Screening tests to identify latent or subclinical *M*. *leprae* infections are warranted to improve the efficacy of post-exposure prophylaxis [52]. Implementing routine skin examinations for foreign workers or employees is still an important policy to support early leprosy diagnosis in our country. Post-exposure prophylaxis may be considered in possible household and workplace contracts.

A limitation of our study was the small sample size as Taiwan is a non-endemic area for leprosy; indeed, most patients were foreigners. Moreover, this study did not include all leprosy patients in Taiwan.

## 5. Conclusions

As studies regarding leprosy have not been updated in Taiwan, a retrospective study was conducted at our hospital, providing clinical information on manifestations, classification, pathologic examination, and drug resistance rates. Notably, our study found a lower drug resistance rate to dapsone than that reported by WHO from 2009 to 2015, indicating that leprosy was well-controlled. Routine examination of drug-resistant genes is not recommended; however, molecular detection of leprosy may be useful in this area, where most patients have indeterminate leprosy. We also compiled current research on diagnostic methods, treatment options, COVID-19 pandemic management, vaccination, and medication for post-exposure prophylaxis in recent studies to assist physicians in the diagnosis and control of leprosy. Bridging the gap between early diagnosis and prevention is still a key step for the eradication of leprosy.

## Figures and Tables

**Table 1:** Summary of patient demographics and clinical presentation.

Demographics and Clinical Presentation	*n*	%
Total patients	28	
Age (in years)		
Mean	39 ± 13 (23–70)	
Sex		
Male	10	35
Female	18	65
Nationality		
Indonesian	15	54
Filipino	6	21
Taiwanese	5	18
Myanma	1	4
Unknown	1	1
Lesion number		
Multiple	24	86
Single	4	14
Clinical classification		
Indeterminate	2	7
Lepromatous	5	18
Borderline lepromatous	11	39
Borderline borderline	3	11
Borderline tuberculoid	5	18
Tuberculoid	1	4
Histoid	1	4

**Table 2:** Summary of pathological features.

Features (*n* = 34)	*n*	%
Granuloma		
+	26	76
−	8	24
Vasculitis		
+	3	9
−	31	91
**Neuritis**		
+	22	65
−	12	35
Necrosis		
+	2	6
−	32	94
Inflammatory infiltrates		
Lymphocyte	15	44
Foamy histiocyte	5	15
Neutrophil	2	6
Plasma cell	7	20
Acid-fast stain		
+	25	74
−	9	26
S100		
+	12	35
Not performed	22	65
Giant cell		
+	2	6
−	32	94
*Mycobacterium leprae* PCR		
Not performed	25	74
+	4	12
−	5	15
Drug-resistant gene		
*folP1* (dapsone)	1	3
*rpoB* (rifampin)	0	0
*gyrA* (ofloxacin)	0	0

+ refers to features that were present; − refers to features that were not present.

## Data Availability

The data that support the findings of this study are available from the corresponding author, P.-F.H., upon reasonable request.

## Supplementary Materials

The following supporting information can be downloaded at https://www.mdpi.com/article/10.3390/diagnostics13243655/s1, Table S1: Patient demographic and clinical characteristics; Table S2: Patient demographic and pathologic characteristics; Table S3: Comparison of patient characteristics between before and during the COVID-19 pandemic.
