# Supplementary material for: Diagnosis and Treatment of Leprosy in Taiwan during the COVID-19 Pandemic: A Retrospective Study in a Tertiaty Center
Source: Diagnostics (Basel). 2023 Dec 13;13(24):3655. doi: 10.3390/diagnostics13243655 (PMC10742743; doi:10.3390/diagnostics13243655)
Supplement: Supplementary file 1 [file diagnostics-13-03655-s001.zip › diagnostics-2675782-SI.pdf]

Table S1. Patient demographic and clinical characteristics

Case No.	Year	Age	Sex	Ethnicity	Type	Reaction	Bacterial Index (L/R ear)	Neural symptoms	Systemic symptoms	Treatment	Treatment duration (month)	Treatment response	complication
1	2009	35	F	Indonesia	BL	NA	0/0	numbness	-	MDT	1*		NA
2	2011	48	M	Myanmar	BB	II	1+/2+	sensory loss/ numbness/ muscle atrophy/ drop hand	Fever	MDT/steroid Thalidomide	24	complete	Type II reaction
3	2011	28	F	Indonesia	BB	NA	5+/5+	numbness	-	MDT	1*		NA
4	2012	32	F	Indonesia	L	NA	1+/0	-	Fever	MDT/Rifampin Moxifloxacin minocycline	30	complete	Vomit nausea
5	2012	23	F	Foreigner	T	NA	0/0	numbness	-	MDT	2*		NA
6	2012	54	M	Taiwanese	I	NA	0/0	numbness	-	MDT	32	Drop out and return	NA
6	2013	54	M	Taiwanese	BB	NA	0/0	-	-	MDT/Clarithromycin	19		NA
7	2013	28	M	Indonesia	BL	II	1+/1+	-	Fever	MDT/steroid	0*		NA
8	2014	35	F	Phillipine	BL	NA	NA	numbness	-	MDT/steroid	1*		NA
9	2015	23	F	Indonesia	BL	NA	multi-bacillary	Sensory loss	-	NA	1*		NA
10	2015	66	M	Taiwanese	H	NA	2+/1+	Numbness muscle weakness	-	MDT Minocycline Levofloxacin Rifampicin	33	complete	clofazimine pigmentation
11	2015	29	M	Philippine	BL	NA	Multi-bacillary	-	-	MDT	1*		NA

12	2015	32	F	Indonesia	L	III	Multi-bacillary	-	Fever	MDT/steroid	0*		NA
13	2016	32	F	Indonesia	L	NA	0/0	-	-	MDT	0*		NA
14	2016	59	M	Taiwanese	BT	NA	0/0	drop hand	-	MDT	18	complete	NA
15	2017	32	F	Indonesia	BL	NA	1+/1+	sensation change	-	MDT/Rifampin	21	complete/ recurred	NA
16	2019	35	F	Indonesia	BT	I	0/0	-	-	MDT/Rifampin Minocycline	15	complete	NA
17	2019	37	F	Indonesia	BT	NA	0/0	-	-	MDT	4	complete	NA
18	2019	44	M	Philippines	BL	NA	0/2+	numbness	-	MDT/Rifampin Minocycline	12	complete/ recurred	NA
19	2019	34	F	Indonesia	L	NA	0/3+	-	-	MDT/Rifampin	1*		NA
20	2019	70	F	Taiwanese	I	NA	0/0	sensory loss	-	MDT	6	complete	NA
21	2020	28	M	Philippines	BT	I	0/0	-	-	MDT/steroid	1*		NA
17	2020	37	F	Indonesia	BT	I	0/0	-	-	MDT/steroid	18	complete	NA
18	2020	44	M	Philippines	BL	I	0/1+	-	-	MDT/Rifampin minocycline/steroid Clofazimine/ clarithromycin	11	complete	NA
22	2020	31	F	Indonesia	L	I	Multi-bacillary	numbness	-	NA	1*		NA
15	2020	35	F	Indonisia	BL	NA	0/0	numbness	-	MDT/Rifampin	6	complete/	NA
23	2022	52	F	Philippines	BL	II	1+/0	-	Fever	MDT/steroid	1*	recurred	NA
24	2022	23	F	Indonesia	BL	NA	5+/0	numbness	-	MDT	6*		NA

F: female, M: male; *: Incomplete treatment; BL: Borderline lepromatous, BT: Borderline tuberculoid, BB: Borderline, L: lepromatous, T: tuberculoid, H: histoid, I: Indeterminate; MDT: multi-drug therapy

Table S2. Patient demographic and pathologic characteristics

Case No	Year	PCR	Drug resistance	Gene	Granuloma	Neuritis	Vasculitis	Necrosis	AFS	S100	Giant cell	Inflammatory cell
1	2009	NA	NA	NA	+	+	NA	NA	+	+	NA	histiocyte
2	2011	NA	NA	NA	+	NA	NA	NA	+	NA	NA	lymphohistiocyte plasma cells
3	2011	NA	NA	NA	+	+	NA	NA	+	+	NA	lymphohistiocyte
4	2012	NA	NA	NA	+	+	NA	NA	+	NA	NA	fomay histiocytes
5	2012	NA	NA	NA	+	+	NA	NA	+	+	NA	lymphocyte
6	2012	-	NA	NA	+	+	NA	NA	NA	+	NA	lymphocyte
6	2013	NA	NA	NA	NA	+	NA	NA	NA	NA	NA	lymphohistiocyte
7	2013	NA	NA	NA	NA	NA	+	+	+	NA	NA	foamy histiocytes, lymphocyte, neutrophils
8	2014	NA	NA	NA	+	+	NA	NA	+	NA	NA	lymphocyte
9	2015	NA	NA	NA	+	+	NA	NA	+	+	NA	foamy histiocytes, lymphocytes, plasma cells
10	2015	+	Dapsone	folP-P55L	+	NA	NA	NA	+	NA	NA	lymphocyte
10	2015	+	Dapsone	folP-P55L	+	NA	NA	NA	+	NA	NA	lymphohistiocyte plasma cells
11	2015	NA	NA	NA	+	+	NA	NA	+	NA	NA	lymphocytes
11	2015	NA	NA	NA	+	NA	NA	NA	+	NA	NA	lymphocytes, plasma cells
12	2015	NA	NA	NA	+	NA	+	+	+	NA	NA	lymphocyte
13	2016	NA	NA	NA	NA	NA	NA	NA	NA	NA	NA	NA
14	2016	NA	NA	NA	+	+	NA	NA	NA	NA	NA	lymphocytes, plasma cells
15	2017	NA	-	NA	+	+	NA	NA	+	+	NA	mononuclear cell

16	2019	NA	NA	NA	+	+	NA	NA	+	+	NA	epithelioid histiocytes
16	2019	NA	NA	NA	+	+	NA	NA	+	+	NA	epithelioid histiocytes
17	2019	-	NA	NA	+	+	NA	NA	NA	NA	NA	lymphocytes
18	2019	+	NA	NA	NA	+	NA	NA	+	NA	NA	foamy histiocytes, lymphocytes
19	2019	NA	NA	NA	+	NA	NA	NA	+	NA	+	histiocytes, plasma cells
19	2019	NA	NA	NA	+	NA	NA	NA	+	NA	NA	Histiocytes, plasma cells
20	2019	-	NA	NA	NA	+	NA	NA	NA	NA	NA	lymphocytes, plasma cells
21	2020	+	NA	NA	+	+	NA	NA	NA	+	+	giant cell, lymphocytes
17	2020	-	NA	NA	+	+	NA	NA	NA	+	NA	histiocytes, plasma cells
18	2020	NA	-	NA	+	+	NA	NA	+	NA	NA	lymphohistiocyte
18	2020	+	-	NA	+	+	NA	NA	+	NA	NA	lymphohistiocyte
22	2020	+	-	NA	+	+	NA	NA	+	+	NA	foamy histiocytes, lymphocytes, giant cells, and plasma cells
15	2020	-	NA	NA	+	+	NA	NA	NA	+	NA	histiocytes
23	2022	NA	NA	NA	NA	NA	NA	NA	+	NA	NA	lymphocytes, foamy histiocytes, and neutrophils
23	2022	NA	NA	NA	NA	NA	+	NA	+	NA	NA	lymphocytes, foamy histiocytes, and neutrophils
24	2022	NA	NA	NA	NA	NA	NA	NA	+	NA	NA	lymphohistiocyte

PCR: polymerase reaction; AFS: acid fast stain

Table S3. Comparison of patient characteristics between before and during the COVID-19 pandemic

Case no.	During the COVID-19 pandemic	Age (years)	Sex	Type	Reaction	Treatment duration (months)	Recurrence	Neurological symptoms	Systemic symptoms
1	No	35	F	BL	NA	NA	NA	Yes	No
2	No	48	M	BB	II	24	No	Yes	Yes
3	No	28	F	BB	NA	NA	NA	Yes	No
4	No	32	F	L	NA	30	No	No	Yes
5	No	23	F	T	NA	NA	NA	Yes	No
6	No	54	M	I	NA	32	Yes	Yes	No
6	No	54	M	BB	NA	19	NA	No	No
7	No	28	M	BL	II	NA	NA	No	Yes
8	No	35	F	BL	NA	NA	NA	Yes	No
9	No	23	F	BL	NA	NA	NA	Yes	No
10	No	66	M	H	NA	33	No	Yes	No
11	No	29	M	BL	NA	NA	NA	No	No
12	No	32	F	L	III	NA	NA	No	Yes
13	No	32	F	L	NA	NA	NA	No	No
14	No	59	M	BT	NA	18	No	Yes	No
15	No	32	F	BL	NA	21	Yes	Yes	No
16	No	35	F	BT	I	15	No	No	No
17	No	37	F	BT	NA	4	Yes	No	No

18	No	44	M	BL	NA	12	Yes	Yes	No
19	No	34	F	L	NA	NA	NA	No	No
20	No	70	F	I	NA	6	No	Yes	No
15	Yes	35	F	BL	NA	6	No	Yes	No
17	Yes	37	F	BT	I	18	No	No	No
18	Yes	44	M	BL	I	11	Yes	No	No
21	Yes	28	M	BT	I	NA	NA	No	No
22	Yes	31	F	L	I	NA	NA	Yes	No
23	Yes	52	F	BL	II	NA	Yes	No	Yes
24	Yes	23	F	BL	NA	NA	No	Yes	No

M, male; F, female; NA, not applicable; BT, borderline tuberculoid; BB, borderline borderline; BL, borderline lepromatous.
